# Health Care Industry Payments to Editorial Board Members of Major Neurosurgery Journals Between 2017 and 2022

**DOI:** 10.1227/neu.0000000000002934

**Published:** 2024-04-08

**Authors:** Netanja I. Harlianto, Zaneta N. Harlianto

**Affiliations:** * Department of Orthopedic Surgery, University Medical Center Utrecht, Utrecht University, Utrecht, the Netherlands;; ‡ Faculty of Medicine, Anton de Kom University, Paramaribo, Suriname

**Keywords:** Conflict of interest, Editorial board, Industry payments, Neurosurgery journal, Open payments database

## Abstract

**BACKGROUND AND OBJECTIVES:**

Financial conflicts of interest between editorial board members and industry could lead to biases and impartial editorial decisions. We aimed to evaluate the frequency, amount, and characteristics of payments to editorial board members of neurosurgery journals over a 6-year period.

**METHODS:**

In this cross-sectional study, editorial board members were derived from the top 10 neurosurgery journals based on Google Scholar metrics. The Open Payments database by the Centers for Medicare and Medicaid Services was accessed to evaluate industry payments to editorial board members from 2017to 2022. Descriptive analyses were performed on payment data, adjusted for inflation using the consumer price indices.

**RESULTS:**

We included 805 editorial board members. After excluding duplicate names, 342 (53.9%) of 634 had received payments between 2017 and 2022. Eight of 10 journals had more than 50% of editorial board members listed in the Open Payments database. Between 2017 and 2022, the total number of payments to editorial board members was $143 732 057, encompassing $1 323 936 in research payments, $69 122 067 in associated research funding, $5 380 926 in ownership and investment interests, and $67 905 128 in general payments. General payments decreased from $13 676 382 in 2017 to $8 528 003 in 2022. Royalties ($43 393 697) and consulting ($13 157 934) contributed the most to general payments between 2017 and 2022. Four journals had a percentage increase in total payments, whereas general payments decreased for 6 journals.

**CONCLUSION:**

Around 54% of editorial board members of neurosurgical journals received industry payments between 2017 and 2022. We identified journal-specific trends in industry payments and highlighted the importance of transparency and disclosure of financial conflicts of interests for neurosurgery journals.

**ABBREVIATIONS:**
COI: conflict of interest
OPD: Open Payment database.

The Open Payment database (OPD) was established in 2014 as part of the Physician Payments Sunshine Act under section 6002 of the Patient Protection and Affordable Care Act of 2010 to foster transparency between physicians and the medical industry. Managed by the Center for Medicare & Medicaid Services, the OPD provides insight into payments from the medical industry to physicians, relationships which may be seen as a potential conflict of interest (COI).^1,2^ The term “medical industry” encompasses pharmaceutical and medical device manufacturers, companies that may rank among the most profitable companies worldwide. For example, the cumulative revenue of the top 35 pharmaceutical companies from the S&P 500 Index totaled $11.5 trillion between 2010 and 2018.^3^ The presence of COIs in medicine may influence how research is conducted, affect medical decision-making, and influence the perception of the public on medicine and science.^4-7^ Publications in medical journals are regarded as objective and valid sources of scientific knowledge. In this study, editorial board members play an important role in the scientific process, making decisions on what research is published.^8^ Moreover, editors have an important role for upholding integrity in research and science publications.^8,9^ Financial COIs between editorial board members and industry may or may not lead to biases and impartial editorial decisions, whether conscious or unconscious. Hence, to minimize this uncertainty, it is important that these COIs are disclosed for transparency.^10^

Within the field of neurosurgery, previous work has aimed to report industry payments to neurosurgeons as reported in the OPD.^11,12^ A previous study between 2014 and 2018 found extensive ties between medical device and pharmaceutical companies and United States–based academic neurosurgeons, as approximately 95% had received payments from industry. These payments increased over time, totaling $266 407 458.^11^ Moreover, approximately $106.77 million in industry research funding was paid out to neurosurgeons in the same period.^12^ To our best knowledge, it is unknown to what extent editorial board members of leading neurosurgical journals receive payments from industry. The aim of this study was to report the frequency and characteristics of industry payments to editorial board members of neurosurgery journals. Furthermore, we aimed to evaluate how these payments have changed over time between 2017 and 2022.

## METHODS

### Data Sources and Data Extraction

This study was performed in accordance with the Strengthening the Reporting of Observational Studies in Epidemiology guidelines for cross-sectional studies.^13^ We identified the top 10 neurosurgery journals according to Google Scholar metrics on December 1, 2023. The Google Scholar journal ranking is based on the highest h5-index (the h-index for all published articles in the past 5 years, ranging from 2018 to 2022) and the h5-median (the median number of citations on which the h5-index is based). The following neurosurgery journals were included: Journal of Neurosurgery; World Neurosurgery; Neurosurgery; Neurosurgical Focus; Journal of Neurosurgery: Spine; Neurosurgical Review; Acta Neurochirurgica; Journal of Neurosurgery: Pediatrics; Operative Neurosurgery; and Neurosurgery Clinics.

All editorial board members listed on the journal website were identified, with the exclusion of administrative assistants and emeritus editors. We ensured that editorial board members who held positions at multiple neurosurgery journals were only included once in the main analyses. The names listed on the OPD were verified using the full name, specialty, and location demographics as provided on the journal website and if needed on the website of their institution. As the online OPD data are publicly available, institutional review board approval was not required for our study.

The OPD lists research payments, associated research funding, ownership and investment interests, and general payments.^14^ For research payments, associated research funding, and ownership and investment interests, each company or corporation was identified with each corresponding payment. We summarized all payments stratified by company or corporation for all 3 categories. General industry payments were further divided into “acquisitions”; “consulting”; “charitable contributions”; “honoraria”; “food and beverage”; “travel and lodging”; “grant”; “gift”; “education”; “entertainment”; “royalty or license”; “current or prospective ownership”; “compensation for serving as faculty or as a speaker for an accredited or unaccredited medical education program”; “compensation for services other than consulting, including serving as faculty or as a speaker at an event other than a continuing education program.” ^14^ Monetary values were adjusted for inflation in 2022 US dollars by means of the US Consumer Price Index data from the US Bureau of Labor and Statistics.^15^

We searched the website of each included journal whether financial COI disclosures were available for individual editorial board members. Moreover, journal websites and publisher's COI policies were evaluated for information on how editorial COIs are handled.

### Statistical Analysis

Statistical analysis was performed using R, version 4.1.3 (R Foundation for Statistical Computing). Data were described using median ± IQR for continuous data and frequency (%) for categorical data. We calculated proportions of the editorial board members who received payments for the total group and for each journal. Trends across time were evaluated for each journal, and the overall payment and payment types for all included members were calculated between 2017 and 2022.

### Data Availability

Available on reasonable request from the corresponding author.

## RESULTS

### Included Journals

A total of 805 individuals listed on the editorial boards of the 10 neurosurgical journals were included. Journal impact factors ranged from 1.9 for Journal of Neurosurgery: Pediatrics to 4.8 for Neurosurgery. The journal with the most individuals listed on the editorial board was Neurosurgery, whereas Neurosurgical Focus and Neurosurgical Clinics had the lowest number listed (Table 1). One hundred seventy-one individuals were listed as editorial board members for 2 or more journals. Of all unique editorial board members, 342 (53.9%) of 634 had received payments between 2017 and 2022 in the OPD. The percentage of editorial board members receiving industry payments ranged from 16.7% (Acta Neurochirurgica) to 100% (Neurosurgery Clinics), median percentage for all journals 68.9%. Eight of 10 journals had more than 50% of editorial board members listed in the OPD.

**TABLE 1.** Included Journals and Percentage Receiving Industry Payments

Journal	Impact factor	No. of individuals listed on editorial board	No. of individuals receiving payments	Percentage of individuals receiving payment
Journal of Neurosurgery	4.1	31	23	74.2
World Neurosurgery	2.0	131	67	51.1
Neurosurgery	4.8	251	159	63.3
Neurosurgical Focus	4.1	3	2	66.7
Journal of Neurosurgery: Spine	2.8	25	20	75
Neurosurgical Review	2.8	54	17	31.5
Acta Neurochirurgica	2.4	114	19	16.7
Journal of Neurosurgery: Pediatrics	1.9	26	20	76.9
Operative Neurosurgery	2.3	169	120	71.0
Neurosurgery Clinics	2.6	2	2	100

### Total Payments

**FIGURE 1.**
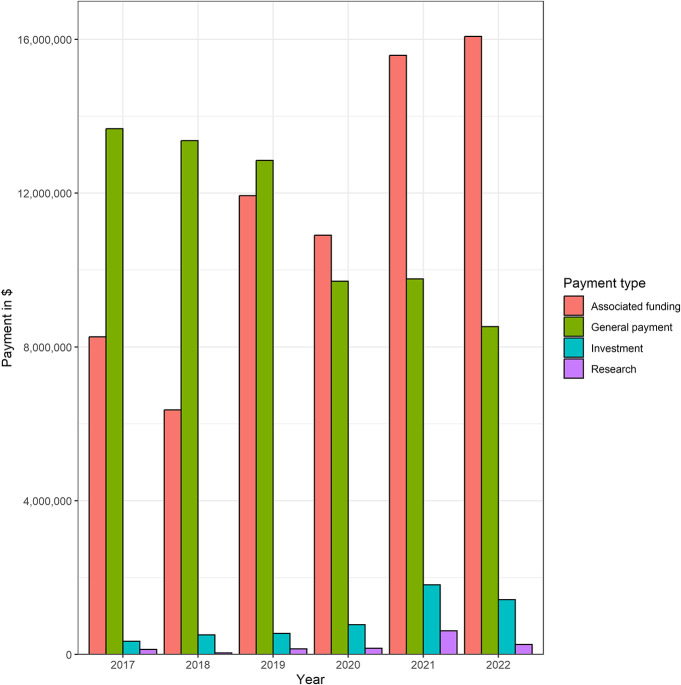
Total payments between 2017 and 2022 by payment type.

**FIGURE 2.**
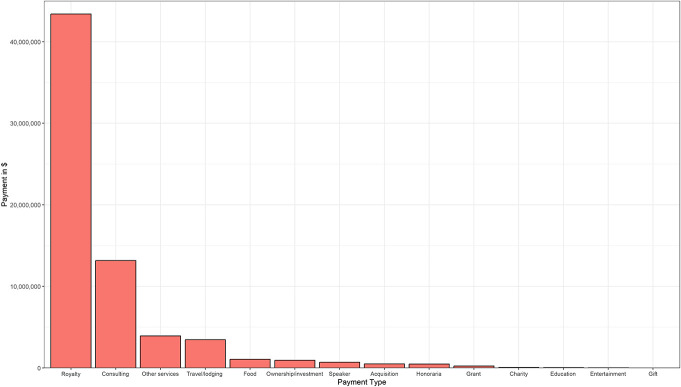
Breakdown of total general payments between 2017 and 2022.

Between 2017 and 2022, the total amount of payments to editorial board members was $143 732 057, encompassing $1 323 936 in research payments, $69 122 067 in associated research funding, $5 380 926 in ownership and investment interests, and $67 905 128 in general payments. Total payments increased between 2017 and 2022 because of an increase in research payments ($128 430 in 2017 vs $252 472 in 2022), associated research funding ($8 261 185 in 2017 to $16 077 468 in 2022), and ownership and investment interests ($336 488 in 2017 vs $1 420 920 in 2022). For the total group, general payments decreased from $13 676 382 in 2017 to $8 528 003 in 2022 (Table 2). A summary of each payment by year is shown in Figure 1. Table 3 shows the top 10 highest paying companies or corporations for research payments, associated research funding, and ownership and investment interests. For research payments, the highest payments were from MicroVention Inc ($395 870), InSightec, Ltd ($395 775), and Nevro Corp ($95 901). The highest associated research funding payments were from Abbott Laboratories ($14 676 084), Merck Sharp & Dohme LLC ($9 207 606), and MicroVention Inc ($4 746 592). For ownership and investment interests, these were Nanovis LLC ($1 272 208), Phasor Health LLC ($1 261 375), and Imperative Care Inc ($794 882). Royalties (total payments: $43 393 697) and consulting (total payments: $13 157 934) contributed the most to general payments between 2017 and 2022. This was followed by travel and lodging (total payments: $3 459 694), other services (total payments: $3 924 144), and food and beverage (total payments: $1 061 985), whereas the lowest total amount was attributed to gifts (total payments: $868). The total amount of payments between 2017 and 2022 stratified by category is shown in Figure 2.

**TABLE 2.** Total Dollar Amount for Each Year

Year	Total payment	Research payment	Associated research funding	Ownership and investment interests	General payment
2017	$22 402 485	$128 430	$8 261 185	$336 488	$13 676 382
2018	$20 270 308	$32 747	$6 363 460	$505 933	$13 368 168
2019	$25 471 153	$141 212	$11 932 277	$543 301	$12 854 364
2020	$21 532 896	$156 477	$10 902 716	$767 050	$9 706 654
2021	$27 776 349	$612 598	$15 584 961	$1 807 234	$9 771 557
2022	$26 278 863	$252 472	$16 077 468	$1 420 920	$8 528 003

**TABLE 3.** Top 10 Highest Paying Companies Between 2017 and 2022

No.	Research payments		Associated research funding		Ownership and investment interests	
	Company	Amount in $	Company	Amount in $	Company	Amount in $
1	MicroVention Inc	$395 870	Abbott Laboratories	$14 676 084	Nanovis LLC	$1 272 208
2	InSightec, Ltd	$395 775	Merck Sharp & Dohme LLC	$9 207 606	Phasor Health LLC	$1 261 375
3	Nevro Corp	$95 901	MicroVention Inc	$4 746 592	Imperative Care Inc	$794 882
4	Arbor Pharmaceuticals Inc	$92 422	Medtronic	$3 732 539	Innovative Surgical Designs Inc	$648 699
5	NICO Corporation	$68 115	InSightec, Ltd	$3 115 796	Aegis Spine Inc	$400 992
6	Sequent Medical Inc	$63 461	GlaxoSmithKline LLC	$2 705 079	Triad Life Sciences Inc	$231 120
7	Bracco Diagnostics	$61 326	Medical Device Business Services Inc	$2 615 511	Smarter Devices LLC	$181 506
8	Functional Neuromodulation Inc	$51 626	Sunovion Pharmaceuticals Inc	$2 450 662	ClearpointNeuro	$149 040
9	Stryker Corporation	$15 158	AstraZeneca Pharmaceuticals	$2 427 022	Ablative Solutions Inc	$127 665
10	ACADIA Pharmaceuticals	$11 821	AbbVie Inc	$1 846 598	DuraStat LLC	$100 000

A detailed breakdown of each category within general payments for each year is shown in **Supplemental Digital Content 1**, **Supplemental Table 1** (http://links.lww.com/NEU/E197) for total payments and **Supplemental Digital Content 1**, **Supplemental Table 2** (http://links.lww.com/NEU/E197) for median payments. Total general payments decreased for 2020 and 2021 for food and beverage ($194 778 in 2017 vs $90 524 in 2020), travel and lodging ($693 525 in 2017 vs $230 335 in 2020), speaker fees ($200 969 in 2017 vs $30 528 in 2020), and other services ($864 992 in 2017 vs $406 783 in 2020). In 2022, these general payments increased for food and beverage ($204 999), travel and lodging ($533 436), and other services ($561 588), though not to a level similar as observed in 2017. Royalty fees on the other hand, decreased over time ($9 708 560 in 2017 vs $4 105 682 in 2022).

### Percentage Change by Journal

The change in total payments and general payments for each journal is shown in Table 4. Between 2017 and 2022, 4 of 8 journals had a percentage increase in total payments (Neurosurgical Focus and Neurosurgery Clinics excluded), the highest for Journal of Neurosurgery ($1 127 386-$7 036 868; 524%) and Journal of Neurosurgery: Pediatrics ($81 376-$584 488; 618%). By contrast, decreases were observed for Neurosurgery ($13 662 777-$11 836 582; −13.3%), Neurosurgical Review ($435 909-$427 445; −1.9%), Operative Neurosurgery ($9 552 367-$7 643 058; −19.9%), and Acta Neurochirurgica ($350 278-$217 049; −39.8%).

**TABLE 4.** Overall Payments Change for Each Journal Between 2017 and 2022

Journal	Total payments in 2017	Total payments in 2022	Percentage change	General payments in 2017	General payments in 2022	Percentage change
Journal of Neurosurgery	$1 127 386	$7 036 868	524	$351 555	$853 263	143
World Neurosurgery	$240 605	$260 290	8.1	$240 605	$258 645	7.5
Neurosurgery	$13 662 777	$11 836 582	−13.3	$7 591 114	$3 111 088	−59
Journal of Neurosurgery: Spine	$2 411 626	$2 920 229	17.4	$2 047 746	$1 973 352	−3.6
Neurosurgical Review	$435 909	$427 445	−1.9	$260 353	$245 619	−5.7
Acta Neurochirurgica	$350 278	$217 049	−39.8	$300 215	$192 525	−35.9
Journal of Neurosurgery: Pediatrics	$81 376	$584 488	618	$70 928	$15 490	−78.2
Operative Neurosurgery	$9 552 367	$7 643 058	−19.9	$6 990 300	$4 721 392	−32.4

Neurosurgical Focus and Neurosurgery Clinics were excluded because the number of editors was <5.

General payments decreased for 6 of 8 journals between 2017 and 2022 ranging from −3.6% to −78.2%, whereas general payments increased for World Neurosurgery ($240 605-$258 645; 7.5%) and Journal of Neurosurgery ($351 555-$853 263; 143%)

Table 5 displays the median payment change between 2017 and 2022. For total payments, median payment change increased for Neurosurgery (23.5%), Operative Neurosurgery (35.9%), Journal of Neurosurgery: Spine (162%), Acta Neurochirurgica (129%), and Journal of Neurosurgery: Pediatrics (284%). Median general payments decreased between 2017 and 2022 for 4 of 8 journals: World Neurosurgery (−9.4%), Operative Neurosurgery (−6.4%), Neurosurgery (−33.1%), and Journal of Neurosurgery (−76.4%).

**TABLE 5.** Median Payment Change for Each Journal Between 2017 and 2022

Journal	Median total payments in 2017	Median total payments in 2022	Percentage change	Median general payments in 2017	Median general payments in 2022	Percentage change
Journal of Neurosurgery	$15 451	$6671	−56.8	$10 830	$2552	−76.4
World Neurosurgery	$1410	$1354	−4.1	$1410	$1277	−9.4
Neurosurgery	$10 357	$12 787	23.5	$3953	$2643	−33.1
Journal of Neurosurgery: Spine	$33 526	$87 991	162	$14 585	$51 645	254
Neurosurgical Review	$17 504	$5802	−66.9	$2346	$3603	53.6
Acta Neurochirurgica	$5900	$13 527	129	$4436	$7062	59.2
Journal of Neurosurgery: Pediatrics	$256	$982	284	$187	$242	29.4
Operative Neurosurgery	$7307	$9929	35.9	$3976	$3723	−6.4

Neurosurgical Focus and Neurosurgery Clinics were excluded because the number of editors was <5.

### Journal Policies and Editor COI

None of the 10 included neurosurgery journals listed individual COIs for each editorial board members. Publisher policies for handling editorial COIs are listed in Table 6. All included journals had online policies or descriptions available how editorial COIs should be managed.^16-19^

**TABLE 6.** Individual Editor and Publisher COI Policies

Publisher	Journal	Individual editor COI available	Publishers' COI policy for editors
The Journal of Neurosurgery Publishing Group	Journal of Neurosurgery	No	“Reviewers and Editors are required annually to submit COI statements to the The Journal of Neurosurgery Publishing Group. In addition, they must disclose if they have a relationship (financial or otherwise) with a manufacturer, researcher, or institution that could predispose them to regard an individual manuscript favorably or unfavorably. When a clear COI exists, the Reviewer or Editor will not be assigned to that manuscript or will recuse themselves from reviewing the manuscript.”^16^
	Journal of Neurosurgery: Spine	No	
	Neurosurgical Focus	No	
	Journal of Neurosurgery: Pediatrics	No	
Wolters Kluwer	Neurosurgery	No	“The journal asks all reviewers (or editors) who suspect they have a COI related to a submission which they are invited to review to contact the Editorial Office. If the reviewer (or editor) is indeed found to have a conflict, the reviewer (or editor) is recused and uninvited.”^17^ Those engaged in the review process should fully disclose financial and other personal interests in an effort to reduce bias and potential conflict in analysis and decision-making. (derived from reviewer invitation letter).
	Operative Neurosurgery	No	
Springer	Neurosurgical Review	No	“Editorial Board Members, Guest Editors and Editors are required to declare any competing interests and may be excluded from the peer review process if a competing interest exists.”^18^
	Acta Neurochirurgica	No	
Elsevier	World Neurosurgery	No	“Any potential editorial COI should be declared to the publisher in writing prior to the appointment of the editor, and then updated if and when new conflicts arise. The publisher may publish such declarations in the journal. The editor must not be involved in decisions about papers which s/he has written him/herself or have been written by family members or colleagues or which relate to products or services in which the editor has an interest.”^19^
	Neurosurgery Clinics	No	

COI, conflict of interest.

## DISCUSSION

In this cross-sectional study, we aimed to identify the extent and frequency of industry payments to editorial board members of leading neurosurgery journals between 2017 and 2022. We found that 53.9% of individuals listed on the editorial board had received payments from industry between 2017 and 2022, totaling $143 732 057. Eight of 10 journals had more than 50% of editorial board members listed in the OPD. Over time, research payments, associated research funding, and ownership and investment payments increased between 2017 and 2022. By contrast, general payments to editorial board members decreased within the same time frame. This decrease was specifically observed from 2020 to 2022, which may be attributed to the COVID-19 pandemic. We observed that speaker fees, food and beverage, and travel and lodging payments for 2020 and 2021 were lower compared with 2017, which slightly increased again for the year 2022. This decline in payment during the COVID-19 pandemic was also observed for the field of radiology.^20^

Industry payments to editorial board members are prevalent across various fields,^21-25^ and our results show that the field of neurosurgery is no exception. Samuel et al^21^ evaluated editorial board member payments from industry for orthopedic surgery for 15 orthopedic surgery journals between 2014 and 2019. They found that 47% of editorial board members received industry payments. Moreover, the total amount of general payments increased for most of the journals over time, a trend which is in contrast to our study results. Kwee and Kwee^22^ assessed the editorial boards of the top 15 United States–based imaging-related journals, for which 41% of editorial board members had received industry payments in 2020. Around 30% of editorial board members for emergency medicine had a financial COI, which was much higher for the field of pathology (70%).^24,25^

In the previous study by Vanood et al,^12^ industry-sponsored research payments between 2014 and 2018 were made to 731 neurosurgeons, for which neurosurgeons affiliated with teaching institutions received a larger proportion of research payments. In their results, most research payments were paid out by Medtronic, Novartis, Pfizer, Stryker, and Sunovion Pharmaceuticals,^12^ whereas in our series of editorial board members, the top 3 paying companies for research payments were MicroVention, InSightec, and Nevro Corp.

Previous work set out to evaluate the public perception on COI regarding industry-funded research. The authors concluded that the general consensus was that industry-sponsored research is beneficial for patients, and therefore, physicians should be involved, irrespective of the funding source.^26^

Collaboration between health care and industry is essential for the advancements in science and improved patient care.^10^ Editorial board members are usually academic neurosurgeons who may hold leading positions and are usually at the forefront of innovation and research. This notion is reinforced by our findings because the proportion of general payments was similar to the amount received for associated research funding in our study at 47% and 48%, respectively. By contrast, between 2014 and 2018 among all academic neurosurgeons, general payments made up of more than 77% of the total amount, whereas the proportion of associated research payments was much lower at 17%.^11^

Much attention has been given to individual authors and their COI, which has been less apparent for editorial COIs.^27^ A former study evaluated disclosure requirements and COI policies of leading neurosurgery journals in 2016. Of the 20 included journals, 89.5% had COI policy statements available for authors.^28^ By contrast, only 26% of included journals had journal policies available on how editor's COI were handled.^28^ None of the included journals had data available on individual editor COI in our study, although COI policies of publishers do address how cases should be handled should an editor have a COI in relation to submitted manuscripts. For example, the Journal of Neurosurgery requires that reviewers and editors submit a disclosure statement each year and that editors and reviewers are not involved in the peer review process or editorial decision-making in the presence of a COI.^16^

Editorial decision-making may be influenced in various ways should a financial COI exist. For example, if a board member receives grant funding from a certain organization, this could persuade them to publish data from that organization; alternatively, if data are supplied that contradicts the information the board member may be investigating on their behalf, this could discourage them from publishing the information. Thus, it is important for neurosurgical journals to disclose COI and to be transparent about their handling of potential COI for editorial board members. Furthermore, disclosing COI is also important to exclude the influencing of study results by industry funding. Efforts by Staartjes et al^29^ evaluated potential COI across 129 neurosurgical randomized controlled trials. No significant association was found between positive study results and COIs.

Our study is the first to evaluate financial COI derived from the OPD for editorial board members of leading neurosurgical journals.

### Limitations

Our study limitations should however be mentioned. Because the OPD only reports on physicians within the United States, we could not report on data from editorial board members from other countries in our analyses. To the best of our knowledge, no database exists that systematically tracks industry payments to non–United States physicians and editorial board members. Second, previous work has established that the OPD contains some inaccuracies for the field of neurosurgery.^30^ We tried to minimize misclassification by using demographics of journal and institutional websites. Finally, we could not ascertain whether editorial decisions were influenced by the findings of industry payments in our study. To our best knowledge, no formal study has previously evaluated what the extent of financial COIs is on editorial decision-making.

## CONCLUSION

To summarize, we found that approximately 54% of editorial board members of leading neurosurgery journals have received industry payments as listed in the OPD in the past 6 years. Total funding increased over time because of increases in research payments, associated research funding, and investing and ownership interests, whereas general payments decreased, which may be partly attributed to the COVID-19 pandemic. We identified journal-specific trends in industry payments and highlighted the importance of transparency and disclosure of financial COI for neurosurgery journals.

## Supplementary Material

SUPPLEMENTARY MATERIAL

## SUPPLEMENTAL DIGITAL CONTENT

**Supplemental Digital Content 1. Table 1.** The total dollar amount for each category and year. **Table 2.** The median dollar amount for each category and year.

## COMMENTS

The authors present a cross-sectional study analyzing industry payments to editorial board members of leading neurosurgery journals. This paper sought to evaluate the amount, frequency, and characteristics of payments to editorial board members from 2017 to 2022 using the Open Payments Database (OPD) by the Centers for Medicare and Medicaid Services. To further investigate financial conflicts of interest (COI) between editorial board members and industry, the study provides financial data from industry payments that, while not proven, could potentially bias and influence editorial decisions. From 2017 to 2022, the authors identified about 54% of editorial board members across 10 neurosurgery journals received industry payments summing up to over 137 million dollars. Within that time frame, it was noted that general payments decreased while research payments, associated funding, and ownership/investment payments increased. No previous study has analyzed industry payments specifically for editorial board members of neurosurgery journals. While the manuscript could not determine how payments influenced the decisions of editorial board members, it reinforces the importance of disclosing financial COI to exclude the influence of study results from funding provided by industry and maintaining transparency and integrity of scientific data reported in the literature. Overall, the financial data presented and underlying methodology for the overall goal of the study provide value to the literature.

**Ever Hernandez and Jonathan H. Sherman**

*Martinsburg, West Virginia, USA*
