# Supplementary material for: Health Care Industry Payments to Editorial Board Members of Major Neurosurgery Journals Between 2017 and 2022
Source: Neurosurgery. 2024 Apr 8;95(4):816–24. doi: 10.1227/neu.0000000000002934 (PMC11377092; doi:10.1227/neu.0000000000002934)
Supplement: SUPPLEMENTARY MATERIAL [file neu-95-0816-s001.docx]

Supplemental Digital Content

Supplemental Digital Content, Table 1. The total dollar amount for each category and year

| **Year** | **2017** | **2018** | **2019** | **2020** | **2021** | **2022** |
| --- | --- | --- | --- | --- | --- | --- |
| **Consulting** | $1,843,097 | $1,906,541 | $2,538,925 | $2,119,698 | $2,272,723 | $2,476,950 |
| **Honoraria** | $126,963 | $51,580 | $45,084 | $60,622 | $90,765 | $93,762 |
| **Food and Beverage** | $194,778 | $217,316 | $204,878 | $90,524 | $149,490 | $204,999 |
| **Travel and Lodging** | $693,525 | $845,022 | $850,182 | $230,335 | $307,194 | $533,436 |
| **Speaker** | $200,969 | $273,665 | $126,691 | $30,528 | $29,944 | $32,238 |
| **Other services** | $864,992 | $716,894 | $762,645 | $406,783 | $611,242 | $561,588 |
| **Grant** | $29,850 | $25,885 | $12,870 | $84,825 | $75,600 | $1,552 |
| **Royalty** | $9,708,560 | $9,253,813 | $7,591,703 | $6,678,633 | $6,055,306 | $4,105,682 |
| **Ownership and investment interest** | $0 | $64,276 | $717,288 | $0 | $138,564 | $6,600 |
| **Charity** | $0 | $0 | $0 | $0 | $0 | $50,000 |
| **Education** | $11,778 | $11,556 | $2,756 | $4,492 | 1,008 | $6,350 |
| **Gift** | $149 | $59 | $0 | $147 | $494 | $19 |
| **Entertainment** | $1,721 | $1,560 | $1,342 | $68 | $1,150 | $1,220 |
| **Acquisition** | $0 | $0 | $0 | $0 | $38,077 | $45,3607 |

Supplemental Digital Content Table 2. The median dollar amount for each category and year

| **Year** | **2017** | **2018** | **2019** | **2020** | **2021** | **2022** |
| --- | --- | --- | --- | --- | --- | --- |
| **Consulting** | $9,074 ($2,752 - $22,667) | $14,073 ($5,060 - $28,452) | $12,412 ($4,015 – $27,685) | $9,908 ($2,884 – $21,383) | $6,026 ($2,633 - $21,241) | $9,775 ($2,830 - $22,846) |
| **Honoraria** | $2,526 ($2,388 - $8,353) | $1,822 ($1,428 - $4,081) | $2,288 ($1,430 - $4,605) | $2,601 ($566 - $3,676) | $1,620 ($698 - $2,295) | $1,606 ($1,000 - $5,625) |
| **Food and Beverage** | $396 ($159 - $1,072) | $381 ($137 - $390) | $440 ($4,172 – $1,086) | $210 ($68 -$590) | $281 ($123 - $867) | $383 ($146 - $1,071) |
| **Travel and Lodging** | $2,277 ($992 - $7,368) | $2,287 ($888 - $9,016) | $1,949 ($826 - $4,958) | $1,435 ($716 - $3,132) | $2,042 ($578 - $5,271) | $1,824 ($876 - $5,431) |
| **Speaker** | $3,582 ($1,975 - $4,527) | $2,332 ($1,749 - $5,830) | $3,432 ($2,176 - $7,722) | $2,545 ($2,314 - $3,930) | $1,080 ($540 - $4,590) | $1,277 ($727 – $8,609) |
| **Other services** | $8,692 ($2,778 - $17,157) | $10,313 ($1,993 - $17,851) | $11,155 ($5,124 - $26,776) | $3,676 ($1,272 - $6,786) | $5,265 ($1,807 - $14,890) | $6,170 ($2,312 - $18,015) |
| **Grant** | $14,925 ($11,194 - $18,656) | $1,399 ($962 - $4,343) | $12,870* | $42,413 ($38,171 - $46,654) | $37,800 ($29,700 - $45,900) | $356 ($242 - $356) |
| **Royalty** | $53,651 ($22,332 - $387,445) | $50,932 ($15,550 - $298,274) | $59,418 ($25,866 - $325,526) | $52,424 ($16,639 - $271,074) | $66,977 ($14,823 - $181,666) | $60,404 ($20,899 - $186,315) |
| **Ownership or investment interest** | $0 | $64,276* | $358,644 ($265,122 - $452,166) | $0 | $69,282 ($39,123 - $99,441) | $6,600* |
| **Charity** | $0 | $0 | $0 | $0 | $0 | $50,000* |
| **Education** | $94 ($58 - $397) | $82 ($34 - $203) | $193 ($68 - $308) | $363 ($45 - $438) | $70 ($40 - $148) | $99 ($99 - $103) |
| **Gift** | $149* | $59* | $0 | $23 ($19 - $41) | $246 ($216 - $278) | $8 ($6 - $8) |
| **Entertainment** | $147 ($57 - $168) | $780 ($449 - $1,111) | $122 ($90 - $169) | $67* | $142 ($59 - $163) | $105 ($22-$255) |
| **Acquisition** | $0 | $0 | $0 | $0 | $19,038 ($17,165 - $20,912) | $19,012 ($9,521 - $226,789) |

* less than three values available. Data are displayed as median (interquartile range)
